# Examination of categorical approach to symptom assessment: cross-validation of foulds’ Delusions-Symptoms-States Inventory with Korean non-patient and patient groups

**DOI:** 10.1186/1471-244X-13-251

**Published:** 2013-10-08

**Authors:** Samuel Suk-Hyun Hwang, Yeni Kim, Jae Seung Chang, Da Young Yun, Yong Sik Kim, Hee Yeon Jung

**Affiliations:** 1 Department of Addiction, Rehabilitation, and Social Welfare, Eulji University, 212 Yangji-Dong, Sujung-Gu, Seongnam 461-713, Gyeonggi, Korea; 2 Institute of Human Behavioral Medicine, Seoul National University College of Medicine, Seoul 110-744, Republic of Korea; 3 Department of Child Psychiatry, Seoul National Hospital, Seoul 143-711, Republic of Korea; 4 Department of Psychiatry, Seoul National University Bundang Hospital, Seongnam 463-707, Gyeonggi, Republic of Korea; 5 Department of Psychiatry, SMG-SNU Boramae Medical Center, Seoul 156-707, Republic of Korea; 6 Department of Psychiatry and Behavioral Science, Seoul National University College of Medicine, Seoul 110-744, Republic of Korea

**Keywords:** Schizophrenia, Self-report measure, Cross-validation, Delusion, Diagnose, Cluster analysis, Hierarchical class model, Power

## Abstract

**Background:**

Foulds’ Delusions-Symptoms-State Inventory (DSSI) has been purported to be a reliable, systematic categorical measure to assess the patients with schizophrenia according to the degree of illness. However, further cross-validations using other clinical measures and diverse samples from other cultures have not been advanced recently. We aimed to examine the validity of the DSSI hierarchical class model using both Korean non-patient and patient (schizophrenia and depression) groups.

**Method:**

The hypothesis of inclusive, non-reflexive relationships among the DSSI classes was tested. The power of DSSI to detect presence of symptoms was assessed via cross-validation with other clinical measures, and the differences between the clinical features among the DSSI classes were examined using the Minnesota Multiphasic Personality Inventory (MMPI).

**Results:**

The high rate of model conformity (91.1%) across the samples and cross-validation with other criterion measures provided further support for the validity of DSSI.

**Conclusions:**

DSSI is a reliable self-report measure that can be applied to both patient and non-patients to assess the presence and severity of psychiatric illness. Future studies that include more diverse clinical groups are necessary to lend further support for its utility in clinical practice.

## Background

Rich variations in psychopathology of schizophrenia have always presented challenges for clinicians who have predominantly relied on descriptive psychopathology for diagnosing and evaluating schizophrenia. Ironically, most traditional conceptualization and nomenclature of schizophrenia have yielded little room to adequately account for the evolving and shifting phenomenology of the illness. For example, given a history of an 'active phase’ of psychotic symptomatology, a diagnosis of schizophrenia precedes other considerations even in the absence of apparent psychotic features [1]. While subtypes, such as 'in remission’ or 'residual’ could be assigned, their diagnostic reliability, validity, and clinical utility have shown to be largely inadequate and hence have been eliminated in DSM-5 [2].

Characterization of the schizophrenia as either/or dichotomy following the disease entity model prevails in both clinical practice and research. However, a notion of continuum from normality to psychotic illness has been articulated for over a century [3], though it has not been rigorously pursued except by a group of British researchers [1]. The severity of illness can be conceived and is embedded in the hierarchical path along which psychiatric diagnose is usually made - in the ascending order of anxiety and other neuroses, depressive neurosis, manic and paranoid psychoses, schizophrenia, and then organic psychosis [4]. Perceivably, such conceptualization of illness is more likely consider the shifting nature of the illness rather than diagnosis, which may have some important implications for treatment and prevention. Individuals with characteristic attenuated psychotic syndrome are several 100-times more likely to develop psychotic disorder in the next year than the general population and present current mood and anxiety symptoms [5]. Nonetheless, a significant proportion of such individuals are identified and provided with interventions much later to prevent substantial damage [6,7].

On the other hand, categorizing patients according to symptom clusters that reflect the level of illness severity along the hierarchical path may hold distinct advantages over other approaches. For example, dimensional approaches to evaluate the presence and severity of illness may better reflect the heterogeneity of the patients but they present difficulties in establishing the range of normality and inadequacies in accounting for the individual differences in 'symptom complexes’ and their prognostic value [8].

Overall, a taxonomic system that better reflects heterogeneity and fluctuating nature of illness within a continuum may deserve more in-depth examination for its validity and applicability in both clinical practice and research. Accordingly, a self-report measure developed to assess the degree of adverse change in personal functioning and severity of clinical disorder in a hierarchical arrangement has been proposed previously by Foulds and Bedford [9]. The central notion is that starting with those without significant level of illness, or not personally ill (NPI), patients could be categorized according to a four-class hierarchical model of the severity of mental illness as follows: dysthymic states (DS) consisting of manifestations of states of anxiety, depression, and/or elation; neurotic symptoms (NS) involving conversion, dissociation, phobia, compulsion, and rumination; integrated delusion (ID) with delusions of persecution, grandeur, and/or contrition; and delusions of disintegration (DD) representing a single syndromal group corresponding to schizophrenia with considerable extent of personal disintegration as an agent of his/her own feelings, thoughts and actions [10,11]. The central hypothesis of this model is inclusive, non-reflexive relationship among the classes, which simply posits that the membership in the higher class also entails membership in the lower ones and that the converse does not hold. Hence, for example, every patient in ID class has both neurotic symptoms and dysthymic states but not delusions of disintegration [12].

The rate of concordance to the model has been found to reach above 90% in the majority of studies [13], with only a few studies that included narrow diagnostic class, such as chronic inpatients [14] and manic and hypomanic patients [15], showing low percentages of about 73%. The concordance rate of one-month retest by the original authors of the DSSI reached above 90% [16]. Despite such robust findings regarding the validity of the hierarchical model, cross-cultural validation and feasibility of the model have not been actively examined.

In the present study, we examined the validity of the DSSI hierarchical class model using both Korean non-patient and patient (schizophrenia and depression) groups by conducting cross-validations with other clinical measures. We tested the hypothesis of inclusive non-reflexive relationships among the classes, i.e. hierarchical organization according to the severity of illness, assessed the power of the DSSI to detect presence of symptoms via cross-validation with other clinical measures, and examined the differences in the clinical features among the DSSI classes using the Minnesota Multiphasic Personality Inventory (MMPI-2).

## Methods

### Subjects

A total of 298 subjects participated in the study but 3 participants (2 non-patients and 1 schizophrenia patient) were excluded from the analysis because they did not complete the DSSI. Hence, the results were based on 185 non-patients (45.4% male) and 40 patients (30% male) with depression and 70 (50.0% male) with schizophrenia. The clinical sample who was recruited from Boramae Medical Center outpatient clinic met the DSM-IV diagnostic criteria for either schizophrenia or depression as determined by two psychiatrists (YK and HYJ). The non-patient group consisted of the families and friends of hospital staffs and community volunteers. They were all screened by a psychiatrist with the Structured Clinical Interview for DSM-IV Axis I Disorder (SCID-I) for presence of any mental illnesses, but none of them were excluded from the study by this procedure. Informed consent form was received from all subjects prior to any study procedures and this study has been approved by the Ethics Committee of Boramae hospital (06-2008-29).

### Measures

Both non-patients and clinical sample completed the DSSI and the Minnesota Multi-phasic Personality Inventory (MMPI-2). The Hamilton Depression Rating Scale (HAMD-17) [17] was completed only by the clinical sample, and the 8-item remission subscale of the Positive and Negative Syndrome Scale (PANSS) [18] was completed by psychiatrist (HYJ) only for the patients with schizophrenia. Brief descriptions of the scales, the scoring method, and the criteria applied for remission or symptom-free state in this study are as follows:

#### ***DSSI***

The DSSI consists of 84 items all prefixed by 'recently’, emphasizing the respondent’s current state. The inventory contains 12 sets of seven items, each set corresponding to a “personal illness syndrome”, a term favored by Foulds in place of mental illness. The items are scored from zero to three, according to endorsement of the item, degree of distress experienced, the frequency of occurrence or the certainty of belief. A person is categorized as having symptoms if he or she scores four or more on any one or more of the 12 sets [19]. Though it had been suggested that a person scoring four or more on any one or more of the 12 sets can be categorized as having symptoms, at least two symptoms from a particular category was required in order to receive a syndrome group diagnosis to allow for some degree of misreading or misunderstanding by the respondent [13]. Each individual then was diagnosed as belonging to a class of disorder (NPI, DS, NS, ID, or DD) in accordance with the class of the highest level syndromal group to which the patient belonged. When an individual did not conform to the model, e.g. scoring on ID and NS without scoring on DS, the highest scored class was assigned and such nonconformity to the model was noted. The scale was translated into Korean by JSC, examined by a professional Korean linguist for appropriateness of expression, and then back-translated into English by a graduate-level bilingual (SSH).

#### ***MMPI-2***

The Korean version of the MMPI-2 [20] consists of various validity scales and 10 clinical scales. This study did not exclude subjects based on any MMPI-2 validity scales to avoid exclusion of those in the most severe state of illness for a more accurate cross-validation with the DSSI. Among the 10 clinical scales, only 1 (Hs: Hypochondriasis), 2 (D: Depression), 3 (Hy: Hysteria), 4 (Pd: Psychopathic Deviation), 6 (Pa: Paranoia), 7 (Pt: Psychasthenia), 8 (Sc: Schizophrenia), 9 (Ma: Hypomania) were included in the analysis, leaving out 5 (Masculine/femininity) and 0 (Social Introversion), and the cut-off score of t > =65 was applied as indicating presence of significant psychiatric symptom [21]. Hence, if a person does not score above 65 on any of the clinical scales, it was assumed to be indicative of symptom-free status.

HAMD-17: HAMD-17 is an interviewer-administered and rated measure whose scores can range from 0 to 54. It includes items such as overall depression, guilt, suicide, insomnia, problems related to work, psychomotor retardation, agitation, anxiety, and loss of weight and loss of insight. With higher score signifying more severe levels of depression, scores between 0 and 6 indicate a normal person or a symptom-free patient in remission. The Korean version of the HAMD-17 used was found to show high interrater reliability (r = .94, *p* < .001) with internal consistency of .76 (Cronbach’s *α*) in a validation study with 33 inpatients and 70 outpatients diagnosed as major depressive disorder or depressive episode of bipolar I disorder according to the DSM-IV criteria [22].

#### ***PANSS***

Eight PANSS items based on 3 primary symptom domains of psychoticism, disorganization, and negative symptoms (psychomotor poverty) have been rated [23]. These items have been selected by Andreasen et al. [18] to define remission in schizophrenia. Patients must score less than or equal to 3 (mild) on these eight items and this must be maintained for at least 6 months to earn remission status. In this study, however, due to cross-sectional nature of our design, we did not apply the duration requirement. The scale items in this study were derived from the validation study of the Korean version of the PANSS which showed high interrater reliability (positive syndromes subscale = .92, negative syndromes subscale = .86) and test-retest reliability ranging from .89 to .95 [24].

### Analysis

The results were largely analyzed in two steps. First, the feasibility of the DSSI as a reliable self-report instrument for assessment of psychiatric symptoms was examined. This was done by examining the fitness of the hierarchical model of personal illness proposed by Foulds, where the inter-class relationship is a “hierarchical non-reflexive logically inclusive one” [13]. It simply means that any person identified to be in one particular class will also meet the qualifications for all the lower classes but not any of the higher classes. We also examined the distribution of the DSSI classes in each subgroup, i.e. schizophrenia and depression patients and non-patients.

Second, we calculated the power (1- β) of the DSSI, where β signifies the false negative rate (Type I error) or failure to detect the presence of symptoms indicated by other criterion measures. Such would be a case where, for example, a person with schizophrenia who was classified as being NPI on the DSSI presented non-remitted status on the PANSS and HAMD-17 and clinically significant level of score of > = 65 on any of the MMPI-2 clinical scales. In addition to also deriving *α* (Type II error), or false positive rate, the same values were obtained for the MMPI-2 against other measures for a direct comparison with the DSSI. We further examined the validity of Fould’s hypothesis of hierarchical structure model of illness by stratifying the groups according to the five classes and examining the distribution of their mean MMPI-2 clinical scale scores. All statistical procedures were carried out using the SPSS 15.0 (SPSS Inc., Chicago, IL) with statistical significance set at *p* < .05.

## Results

### The fitness of the DSSI hierarchical model

The fitness of the hierarchical model of personal illness proposed by Foulds was examined in light of various demographic and clinical variables and the results are presented in Table 1. The rate of conformity of the data to the proposed hierarchical model reached 91.2% for all subjects. Among the conforming participants, highest conformity was found among those in the age of 30’s and in control group. Within the clinical samples, those with less than 1 year of illness showed the highest conformity, followed by those with more than 10 years of illness.

**Table 1:** Fitness of the hypothesized hierarchical class structure of the DSSI according to gender, age, diagnostic group, and duration of illness (n: % within)

**Gender**	**Male (131: 89.2), Female (164: 92.6)**
**Age**	**Below 20 (16: 81.3), 20–29 (89: 87.6), 30-39 (103: 97.1), 40-49 (45: 88.9), above 50 (40: 90.0)**
**Diagnostic Group**	**Schizophrenia (70: 84.3), Depression (40: 82.5), Non-patient (185: 95.7)**
**Duration of Illness**^**a**^	**Below 1 yr. (27: 92.6), 1–3 yrs. (16: 81.3), 3–5 yrs. (9: 66.7), 5–10 yrs. (17: 70.6), ** **Over 10 yrs. (35: 85.7)**
**Total**	**295: 91.2**

^a^Based on 104 data due to 6 unknown cases.

Presented on Table 2 is the frequency of non-conforming DSSI patterns in each diagnostic group. Among 26 (8.8%) non-conforming participants, 5 (1.7%) scored on NS as their highest class, 14 (4.1%) on ID, and 7 (2.4%) on DD. Only the schizophrenia group showed non-conforming patterns at DD, whereas both non-patient and depression groups showed non-conforming patterns at ID.

**Table 2:** Frequency of non-conforming DSSI patterns according to the diagnostic group

**Assigned class**	**Pattern**	**Non-patient (n = 185)**	**Depression (n = 40)**	**Schizophrenia (n = 70)**	**Total (n = 295)**
**NS**	**(0 1 0 0)**	**2**	**0**	**3**	**5**
**ID**	**(1 0 1 0)**	**3**	**4**	**1**	**8**
	**(0 1 1 0)**	**1**	**1**	**0**	**2**
	**(0 0 1 0)**	**2**	**2**	**0**	**4**
**DD**	**(1 0 0 1)**	**0**	**0**	**1**	**1**
	**(1 1 0 1)**	**0**	**0**	**4**	**4**
	**(1 0 1 1)**	**0**	**0**	**1**	**1**
	**(0 1 0 1)**	**0**	**0**	**1**	**1**
	**Total**	**8**	**7**	**11**	**26**

*DSSI* Delusions-Symptoms-States Inventory, *NS* Neurotic symptoms, *ID* Integrated delusions, *DD* delusions of disintegration.

### The distribution of the DSSI classes according to diagnostic groups

**Figure 1:**
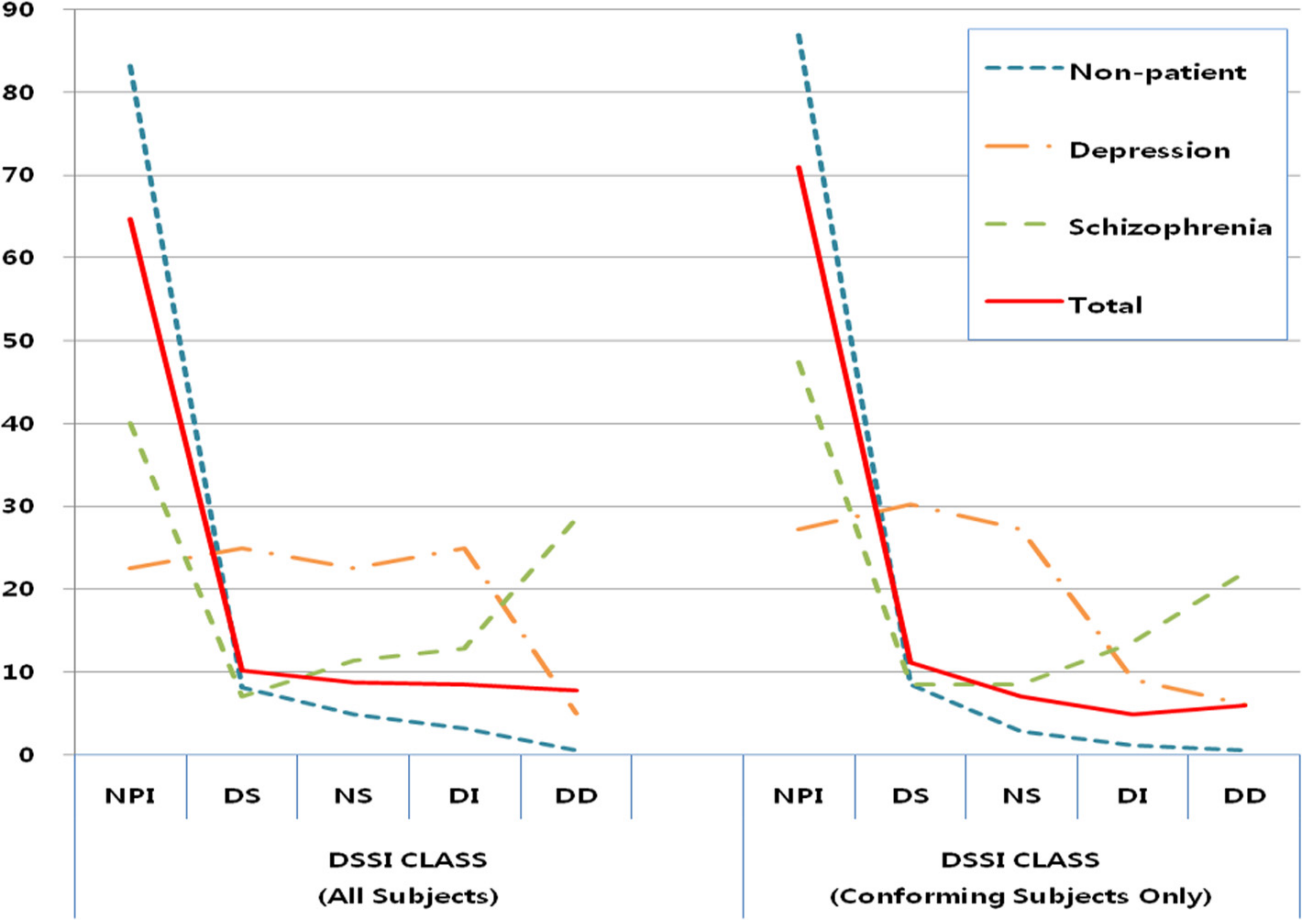
**Distribution of the DSSI classes according to diagnostic groups (% within each group).** DSSI, Delusions-Symptoms-States Inventory; NPI, not personally ill; DS, dysthymic states; NS, neurotic symptoms; ID, integrated delusions; DD, delusions of disintegration.

Presented in Figure 1 is the distribution of the DSSI classes according to diagnostic groups and it includes graphs of all and of only the conforming subjects. Evident in both graphs are the steady decreasing rate of the control and increasing rate of the schizophrenia group with higher classes with symptoms. As for the depression patients, they showed a steady falling trend with higher classes only when the conforming subjects were included.

### Power of the DSSI to detect presence of psychiatric symptoms

Presented on Table 3 is the power (1- β) of the DSSI to detect presence of psychiatric symptoms on other criterion measure for each subgroup based on 265 subjects who completed the MMPI-2 (63 schizophrenia, 36 depression, 166 non-patient). We have also included α (Type II error), or false positive rate. These values are also presented for MMPI-2 against other measures for comparison. Here, regardless of the conformity to the DSSI model, any subjects who scored on any DSSI subscale, i.e. subjects who were not classified as NPI, was regarded as indicating some psychiatric symptom.

**Table 3:** Type I and II error and power of the DSSI against other criterion measures in each subgroup

**Group**	**Measure**	**Criterion**	**Type I error**	**Power**	**Type II error**
			**β**	**(1- β)**	**(α)**
Control	DSSI	MMPI-2	.16	.84	.11
Depression	DSSI	HAMD-17 Remission	.08	.92	.13
		MMPI-2	.08	.92	.17
	MMPI-2	HAMD-17 Remission	.17	.83	.08
Schizophrenia	DSSI	PANSS Remission	.17	.83	.19
		HAMD-17 Remission	.00	1.00	.39
		MMPI-2	.14	.86	.06
	MMPI-2	PANSS Remission	.16	.84	.27
		HAMD-17 Remission	.02	.98	.48
Depression + Schizophrenia	DSSI	HAMD-17 Remission	.03	.97	.29
		MMPI-2	.12	.88	.10
	MMPI-2	HAMD-17 Remission	.05	.95	.34
Total	DSSI	MMPI-2	.14	.86	.11

*DSSI* Delusions-Symptoms-States Inventory, *MMPI-2* Minnesota multiphasic personality inventory, *HAMD-17* Hamilton depression rating scale (17-item), *PANSS* Positive and negative syndromes scale.

The results showed that the DSSI has relatively high levels of power (1- β) for all measures across all groups. The power of the DSSI to detect presence of any psychiatric symptoms in the total sample, non-patient, and clinical subgroups based on MMPI-2 as the criterion measure all reached over .80. Furthermore, the DSSI showed higher levels of power than MMPI-2 in detecting presence of any psychiatric symptoms in depression patients, applying the HAMD-17 remission status criterion. In patients with schizophrenia, the DSSI showed similar levels of power as MMPI-2 in detecting presence of psychiatric symptoms using both HAMD-17 and PANSS remission criteria.

In terms of the Type II error, the DSSI showed the highest level of false positive rate in patients with schizophrenia on HAMD-17 remission criteria, but otherwise false positive rate was kept under .20 for other subgroups and the total group in all measures.

### MMPI-2 clinical scale profiles according to 5 DSSI classes

**Figure 2:**
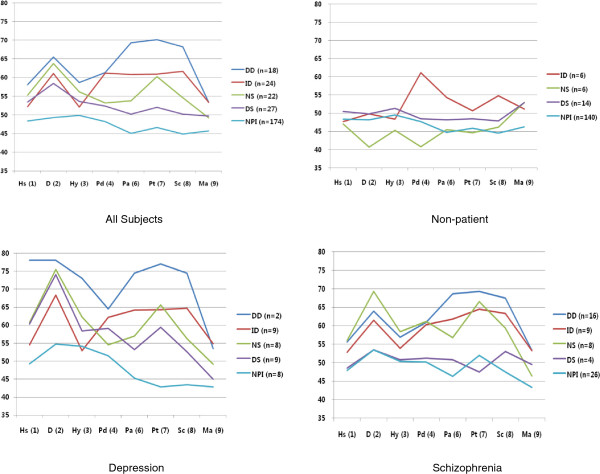
**MMPI-2 clinical scale profiles of 5 DSSI classes in all subjects and according to diagnostic group.** DSSI, Delusions-Symptoms-States Inventory; MMPI-2, Minnesota Multiphasic Personality Inventory; NPI, not personally ill; DS, dysthymic states; NS, neurotic symptoms; ID, integrated delusions; DD, delusions of disintegration. Hs, hypochondriasis; D, depression; Hy, hysteria; Pd, psychopathic deviation; Pa, paranoia; Pt, psychasthenia; Sc, Schizophrenia; Ma, Hypomania.

Presented in Figure 2 are the MMPI-2 clinical scale profiles of the 5 DSSI classes for all subjects (including those who do not conform to the model) and each subgroup.

For all subjects, none of the mean clinical scale scores of MMPI-2 were above 55 for the NPI class. As for the DS class, only the D(2) clinical scale peaked above 55. For the NS class, only D(2) and Pt(7) peaked above the score of 60. For the ID class, D(2), Pd(4), Pa(6), Pt(7), and Sc(8) showed relatively high score peaking above 60, and for the DD class, the mean scores of all clinical scales were highest among the classes but D(2), Pa(6), Pt(7), and Sc(8) in particular reached the clinically significant score of 65.

In case of non-patient group, the NPI class scored slightly lower than the DS class across all clinical scales, and the NS class showed some overlap in scores with the NPI class. The highest scores among the classes were obtained from the ID class in Pd(4), Pa(6), Pt(7) and SC(8) scales, and no one was categorized as DD from this group.

As for depression patients, the NPI class did not have any mean clinical scale score over 55. The DS class showed scores of over 55 on D(2), Hy(3), Pd(4), and Pt(7) clinical scales, but D(2) had the most pronounced peak, being the only scale to reach the clinically significant score of 65. The NS class showed two peaks reaching over the score of 65, i.e. D(2) and Pt(7). As for the ID class, only D(2) peaked over 65, but Pa(6), Pt(7), and Sc(8) were scored closely to 65. There were only 2 in the DD class and collapsing those with those in the ID class would have resulted in raising the mean scores of Pa(6), Pt(7), and Sc(8) to the clinically significant level in addition to D(2) for the combined group.

Lastly, for the schizophrenia subgroup, a large overlap in scores was observed between the NPI and ID classes, with none of the clinical scales reaching scores higher than 55. For the NS class, similar to the depression patients, most scales were above the score of 55, but only D(2) and Pt(7) reached the clinically significant score of 65. The ID class showed Pt(7) and Sc(8) to be the highest scoring subscales right below 65, and the DD class had Pa(6), Pt(7), and Sc(8) scoring well over 65.

The MMPI-2 clinical scale profiles of the 5 DSSI classes that included only those who conformed to the DSSI model did not noticeably differ from the overall trend presented here, except that 1) the ID class in the depression group showed clinically significant mean scores of over 65 on Pd(4), Pt(7), and Sc(8) scales and 2) the NS class in the schizophrenia group also showed mean scores of over 65 on D(2), Pd(4), Pt(7).

## Discussion

In this study, the high rate of model conformity across the samples and cross-validation with other criterion measures provided empirical support for the DSSI hierarchical class organization and demonstrated its potential usefulness as a self-report measure for assessment of presence and severity of symptoms.

Our finding on the DSSI model conformity based on all subjects (91.1%) was consistent with the majority of the past studies [13]. The model conformity of the schizophrenia group (84.3%) was also comparable to those of previous studies including chronic inpatients which ranged from 73.8% to 85.0% [14,25,26]. On the other hand, the conformity rate of our depression group (82.5%) was lower than that of one other study reported on depression group involving only inpatients (92.3%) [27]. Among the nonconforming patients, 71% had duration of illness of longer than 5 years and this seemed to well correspond to the overall decline in the rate of model conformity with chronicity of illness. The nonconforming depression patients were all categorized within DI and there may be a distinct possibility that some of them may have major depression with psychotic features. Such patients have been found to have longer duration of episodes and greater recurrence than those with nonpsychotic depression [28]. Nonetheless, further examination of the relationship between the duration of illness and model conformity is warranted for the depression patients due to a relatively small sample size in our study.

A closer examination of the non-conforming patterns yielded an interesting observation that of 26 non-conforming participants, the overwhelming majority (23 participants including 5 of the NS class) did not report the symptoms of the adjacent subordinate class. Angelopoulos and Economous also reported a comparably high proportion (32 out of 47 non-conforming participants including 7 of the NS class) that did not report the symptoms of the adjacent subordinate class among the inpatients with schizophrenia [11]. Such results suggested that it may be more common for symptoms of one class to overshadow those of the adjacent subordinate class than of any other subordinated classes, which may be an important addition to Foulds’ suggestion that symptomatology from the higher class may mask, confuse, override, or distract from that in the lower classes [29].

The majority of past studies on the distribution of the DSSI classes have been conducted on general psychiatric patients and those involving specific psychiatric groups all dealt exclusively with inpatients except for one study that included day-patients [30]. This was in keeping with the notion that the hierarchical model is applicable only to acute patients [13,26]. Hence, no other study has included a large number of outpatients with chronic schizophrenia, not to mention non-patients – apart from that by the original authors of the DSSI [10]. The results of our study however suggested that the DSSI can be applied as effectively to both subclinical stabilized chronic illness group and non-patients as a screening measure for presence of active symptoms. The sensitivity and specificity of the DSSI in detecting the presence of symptoms among schizophrenia and depression patients was comparable to those of MMPI-2 clinical scales in light of symptom remission criteria of HAMD-17 and PANSS, all of which require more time and trained staff.

Lastly, there are a number of important issues that should be raised prior to generalizing the results of our study. First, our results need to be considered in light of the Berkson’s fallacy: since our non-patient group has been screened with SCID-I, whilst none had been excluded by this process, there is a possibility that the composition of our non-patient group may actually under-represent the individuals with more severe levels of illness [31]. Also, the clinical group was not screened for comorbidity of psychiatric illness, not to mention alcohol dependence, with DSM-IV by the psychiatrists. Furthermore, inclusion of other diagnostic groups, such as anxiety or personality disorders, was not considered in our study design. Since such factors may have significant affect on the conformity of the model, future studies using broader range of diagnose and more refined study design are warranted to lend further support for the validation of the DSSI.

## Conclusions

In summary, the results of the present study provided further support for the utility of the DSSI in covering a relatively wide range of psychiatric symptom spectrum. It may be usefully applied for not only the detection of presence of psychiatric symptoms among non-patient groups but also assessment of alleviation or aggravation of illness in patients. The DSSI can thereby provide complementary information for diagnosis, determination of the treatment efficacy, and decisions concerning maintenance of treatment strategy.

## Abbreviations

D: Depression; DD: Delusions of disintegration; DS: Dysthymic states; DSSI: Delusions-Symptoms-State Inventory; HAMD-17: Hamilton depression rating scale; Hs: Hypochondriasis; Hy: Hysteria; ID: Integrated delusion; Ma: Hypomania; MMPI: Minnesota Multiphasic personality inventory; NPI: Not personally ill; NS: Neurotic symptoms; Pa: Paranoia; PANSS: Positive and negative syndrome scale; Pd: Psychopathic deviation; Pt: Psychasthenia; Sc: Schizophrenia; SCID-I: Structured clinical interview for DSM-IV Axis I disorder.

## Competing interests

The authors declare that they have no competing interest.

## Authors’ contributions

SS-HH was involved in the analysis and interpretation of the data and drafting and editing of the manuscript. YK and CJS were involved in the designing of the study and data collection. DYY was involved in data collection and analysis of the data. YSK and HYJ were involved in the designing of the study and data collection and analysis, as well as in the drafting and editing of the manuscript. All authors read and approved the final manuscript.

## Pre-publication history

The pre-publication history for this paper can be accessed here:

http://www.biomedcentral.com/1471-244X/13/251/prepub
